# Anti-Aging Potential of Substance P-Based Hydrogel for Human Skin Longevity

**DOI:** 10.3390/ijms20184453

**Published:** 2019-09-10

**Authors:** Da Jung Kim, Song Sun Chang, Jungsun Lee

**Affiliations:** Research and Development Institute, Biosolution, Seoul 01811, Korea

**Keywords:** substance P, anti-aging effect, anti-inflammatory effect, skin absorption, 3D human skin model, cosmetic ingredient

## Abstract

Skin aging is generally caused by a decline in the components of the extracellular matrix (e.g., collagen and elastin) and due to inflammatory phenomena. Many growth factors and peptides with cell-growth and collagen-synthesis activities have shown promise in their application in anti-aging materials. However, the effect of collagen production, without anti-inflammatory effect, and skin penetration may not be enough for their use in anti-aging agents. Previously, we reported a substance P (SP)-based hydrogel (SP gel) that had potential wound-healing activities via induction of skin cell regeneration and collagen synthesis. Here, we analyzed the anti-aging activities and skin absorption effects of SP gel to extend its characterization. Toxicity tests, performed on human dermal fibroblasts (HDFs) and on a reconstructed 3D human skin model, indicated SP gel to be safe for long-term use, without causing irritation, even at high concentrations. In-vitro analysis revealed that SP gel elicited stronger collagen production activities than SP alone, and promoted anti-inflammatory effects with increased skin absorption properties. Moreover, SP gel did not induce melanin synthesis in a keratinocyte-melanocyte co-culture system. Together, the results suggest that SP gel has potential cosmetic effects and applicability as a novel ingredient in anti-aging products.

## 1. Introduction

Aging of skin occurs due to various processes, including internal (cellular metabolism, hormonal changes, and genetic mutation) and external factors (toxins, chemicals, and ultraviolet (UV) radiation) [1,2,3]. Aged skin is biologically characterized by a general decline of the components of extracellular matrix (ECM), with disorganized and reduced collagen and elastin [4,5]. Additionally, an inflammatory phenomenon is usually induced in aging skin, causing wrinkling and thickening of the dermis and epidermis. Pro-inflammatory mediators released from inflammatory cells can enhance the activation of collagenases, named matrix metalloproteinases (MMPs), thus leading to collagen degradation [6,7,8].

Many anti-aging materials, possessing the ability to enhance collagen synthesis, have been suggested in recent days. One of the well-known materials is the growth factor. Growth factors have been studied extensively for skin wound healing [9]. Many of them, such as platelet-derived growth factor (VEGF), epidermal growth factor (EGF), and keratinocyte growth factor, directly affect collagen synthesis via a network of inter and intracellular signaling pathways [9,10]. However, topically applied growth factors have not been very helpful as anti-aging agents due to their large molecular size, which limits their ability to penetrate the tight stratum corneum [11]. In addition to growth factors, many anti-aging peptides of low molecular weight have also been developed. For example, palmitoyl tripeptides prevent collagen degradation by interfering with collagen reduction by collagenases, such as MMP1 and 3 [12]. Moreover, copper tripeptides, the most well-examined peptides, have been shown to stimulate procollagen synthesis [13].

Although several studies have indicated the promotion of collagen synthesis by topically applied growth factors and peptides, the single effect of collagen synthesis, without anti-inflammatory effect, may not be enough to clinically improve anti-aging performance. For more obvious effects, the compound needs to be delivered into the deep layers of the skin. Therefore, there is a strong need for the development of new materials, which possess the dual function of collagen synthesis and anti-inflammatory effects, along with increased skin absorption.

Substance P (SP), a small-sized peptide consisting of 11 amino acids, exhibits potential wound healing activity, exerted via induction of cell proliferation, collagen synthesis, and anti-inflammatory effects [14,15,16]. However, the therapeutic application of SP has been limited by its low stability, which can delay its healing properties [17,18,19]. In our previous study, we developed a novel formulation of SP, known as SP gel, which increases the stability of SP under various storage conditions [20]. In addition to its stability, SP gel has shown more effective wound healing than SP alone by enhancing keratinocyte and fibroblast proliferation.

In the current work, we have extended the characterization of SP gel and demonstrated its promotion of collagen synthesis and anti-inflammatory effects for anti-aging performance. Skin absorption and pigmentation by SP gel were also examined further. Our findings demonstrate higher anti-aging potential of SP gel over that of SP alone, with increased skin absorption effect, and hence, broader cosmetic applicability.

## 2. Results

### 2.1. In-Vitro Toxicity of SP Gel

For the SP gel to be of anti-aging application, high activities combined with low toxicity against human skin would be desirable. Therefore, in-vitro toxicity of SP gel was evaluated using human dermal fibroblasts (HDFs) and a reconstructed 3D human skin, keraskin^®^-FT. 

**Figure 1:**
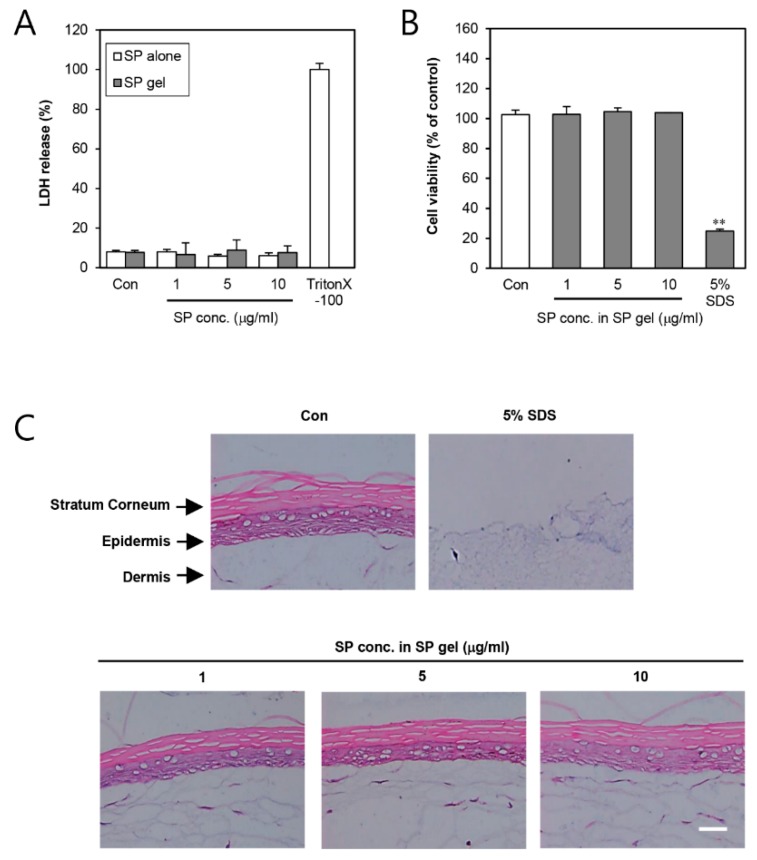
Effect of substance P (SP) gel on the viability of HDFs and in-vitro reconstructed 3D human skin, keraskin^®^-FT. (**A**) For damaging effects of SP gel formulation on cell membrane, HDFs were treated with PBS (Con; control), SP alone, or SP gel (1–10 μg/mL) for 24 h, and cell viability was determined by LDH assay. (**B**,**C**) Skin irritability was tested using SP gel (1–10 μg/mL) on in-vitro reconstructed 3D human skin, keraskin^®^-FT. Tissue viability in the SP gel-treated group was analyzed by MTT assay (B) and histological examination (C), PBS was used as a control. Values represent mean ± SD from three independent experiments. Each value was compared with the control using Student’s t-test (** *p* < 0.01). Scale bar = 500 μm. HDF, human dermal fibroblast; LDH, lactate dehydrogenase; MTT, 3-(4,5-dimethylthiazol-2-yl)-2,5-diphenyl tetrazolium bromide; PBS, phosphate-buttered saline; SDS, sodium dodecyl sulfate.

First, we assessed the damaging effects of SP gel on cell membrane, with respect to that of SP alone, by lactate dehydrogenase (LDH) assay using HDFs (Figure 1A). The LDH assay was further used, in addition to the previous 3-(4,5-dimethylthiazol-2-yl)-2,5-diphenyl tetrazolium bromide (MTT) assay [20], for more accurate toxicity measurement of SP gel. Results showed that neither SP gel nor SP alone showed any cell membrane damage against HDFs at all tested concentrations. Moreover, treatment with a vehicle lacking SP had no effect on the cell membrane relative to medium containing PBS only. To further test the potential irritation on human skin due to the SP gel, we applied the latter, at different concentrations, on the full thickness skin model, keraskin^®^-FT (Figure 1B,C) for a total of 24 h. To compare the effects on tissue viability, 5% sodium dodecyl sulfate (SDS), a known irritant, was used as a positive control. SP gel (1–10 μg/mL) was non-toxic to keraskin^®^-FT, as demonstrated by the MTT assay (Figure 1B), indicating no potential skin irritation compared to control (PBS treatment). In addition, histological examination revealed the keraskin^®^-FT to have suffered no damage at all by SP gel (Figure 1C). The same results were obtained in the group treated with SP alone (Figure S1). Based on these results, even a high concentration of SP gel (10 μg/mL of SP) was concluded to be non-toxic, causing no skin irritation.

### 2.2. Effect of SP Gel on the Production of Collagen

**Figure 2:**
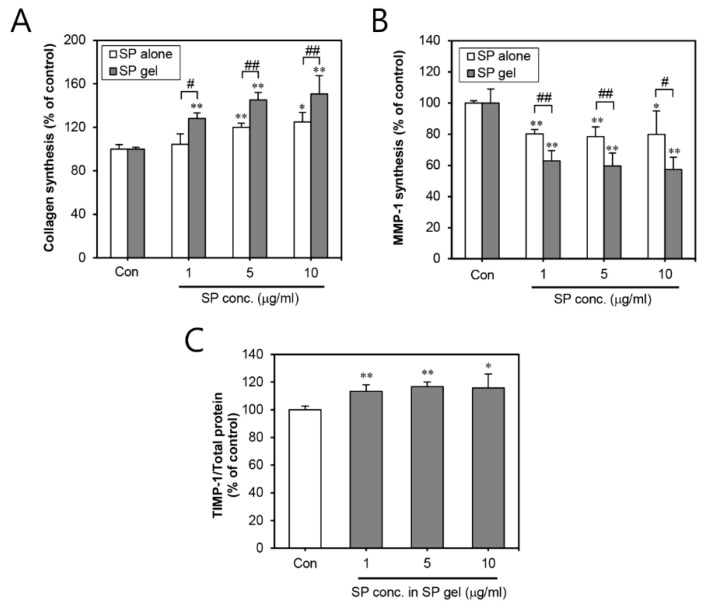
Collagen-increasing effects of SP gel in HDFs. Effects of SP gel on type 1 collagen (**A**), MMP-1 (**B**), and TIMP-1 (**C**) were measured in HDFs. HDFs were treated with PBS (Con; control), SP alone, or SP gel (1–10 μg/mL) for 24 h and the expression of type I procollagen, MMP-1, and TIMP-1 was measured using the Procollagen Type I C-peptide (PIP) EIA kit, human MMP-1 ELISA kit, and TIMP-1 ELISA kit, respectively. PBS was used as a control. Values represent the mean ± SD from three independent experiments. * *p* < 0.05, ** *p* < 0.01 vs. PBS-containing medium without SP; ^#^ *p* < 0.05, ^##^ *p* < 0.01 vs. SP alone. HDF, human dermal fibroblast; PBS, phosphate-buffered saline; MMP-1, matrix metalloproteinase-1; TIMP-1, tissue inhibitors of metalloproteinase-1; ELISA, enzyme-linked immunosorbent assay.

To examine the effect of SP gel on collagen synthesis on the skin, HDFs were treated with SP gel. Our results demonstrated that SP gel, containing 1–10 μg/mL of SP, significantly (*p* < 0.01) increased type I procollagen production to 128.05 ± 5.19, 145.19 ± 6.80, and 150.68 ± 16.70%, compared to the control treatment (PBS) (Figure 2A). Otherwise, as shown in Figure 2A, no noteworthy difference in type I collagen production was observed across HDFs treated with 1 μg/mL of SP alone. While SP alone did have some effect on collagen production at concentrations of 5–10 μg/mL (119.96 ± 3.8 and 124.77 ± 8.87%,), this effect was significantly (*p* < 0.01) lower than with SP gel.

Subsequently, we assessed the levels of MMP-1 associated with collagen degradation in SP gel-treated HDFs. Our results revealed that SP gel containing 1–10 μg/mL of SP significantly (*p* < 0.01) inhibited the MMP-1 levels to 62.88 ± 6.54, 59.54 ± 8.35, and 57.44 ± 7.69%, compared to that in the control HDFs (PBS treatment) (Figure 2B). As expected, SP alone, containing 1–10 μg/mL of SP, also inhibited the levels of MMP-1 to 80.18 ± 2.88, 78.48 ± 6.19, and 79.84 ± 15.08%, although these effects were significantly lower than those of SP gel. In the HDFs treated with SP gel, at SP concentrations of 1–10 μg/mL, the expression levels of tissue inhibitor of metalloproteinase (TIMP)-1, which causes inhibition of MMP-1, were increased to 113.41 ± 4.74, 116.76 ± 3.31, and 115.8 ± 10.13% compared to that in the control treatment (PBS) (Figure 2C). These results together suggested that SP gel can accelerated type I procollagen production by inhibiting MMP-1 and enhancing TIMP-1 expression in the HDFs. Comparative analysis of SP gel and SP alone revealed SP gel to be more effective in inducing collagen production than SP alone.

### 2.3. Efficacy of SP Gel in Skin Inflammation

**Figure 3:**
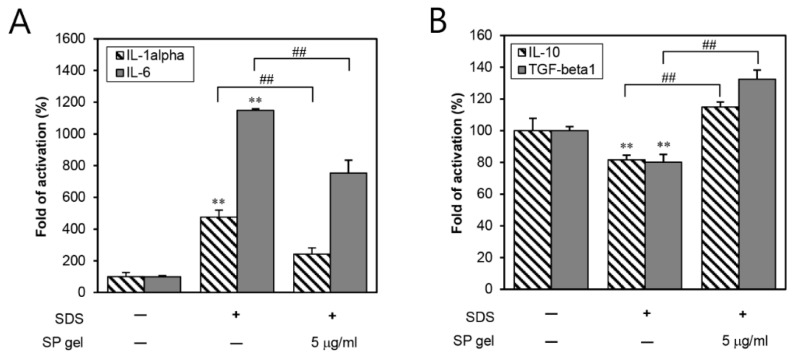
Anti-inflammatory effect of SP gel in SDS-stimulated HEKs. HEKs were simultaneously incubated with 0.0015% SDS and SP gel for 24 h. The cell culture supernatants were harvested and the level of inflammatory factors was measured with ELISA kits. Relative fold increase of pro-inflammatory factors, IL-1 alpha and IL-6 (**A**), and anti-inflammatory factors, IL-10 and TGF-beta 1 (**B**), was detected, as described in the experimental procedures; PBS was used as a control. All values represent the means ± SD from three independent experiments. ** *p* < 0.01 vs. PBS-containing medium without SP; ^##^ *p* < 0.01 vs. only SDS-treated HEKs. HEK, human epidermal keratinocyte; SDS, sodium dodecyl sulfate; ELISA, enzyme-linked immunosorbent assay; PBS, phosphate-buffered saline; IL, interleukin; TGF, transforming growth factor.

To investigate the effect of SP gel on the expression of inflammatory cytokines, we examined whether SP gel had an anti-inflammatory role by decreasing IL-1 alpha or -6 and increasing IL-10 or transforming growth factor (TGF)-beta 1 in SDS-induced human epidermal keratinocytes (HEKs). PBS-treated cells were considered as negative controls while SDS-stimulated cells served as positive controls. As shown in Figure 3A, 24-h treatment with SP gel (5 μg/mL of SP in SP gel) significantly (*p* < 0.01) reduced the SDS-induced expression of IL-1 alpha and IL-6. On the other hand, SP gel significantly (*p* < 0.01) increased the SDS-induced expression of IL-10 and TGF-beta 1 (Figure 3B). These results suggest an anti-inflammatory effect of SP gel on the HEKs. 

### 2.4. Skin Absorption Properties of SP Gel In Vitro

**Figure 4:**
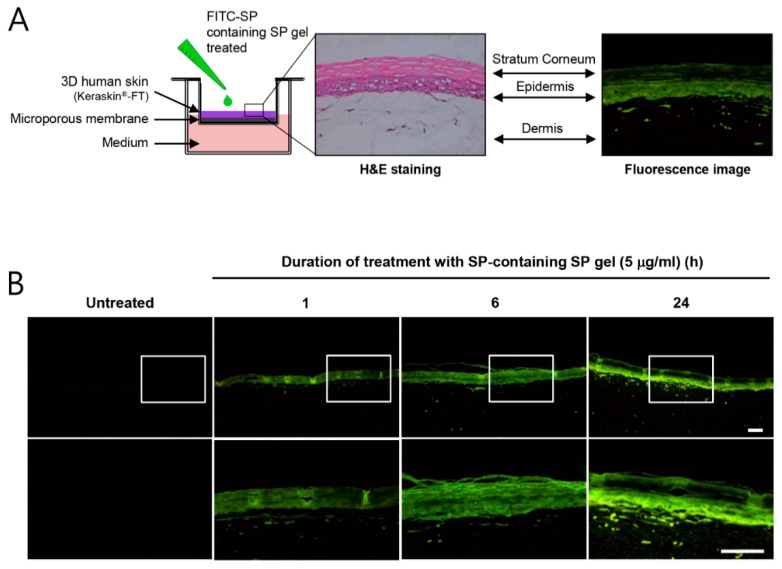
Skin absorption of SP gel into in-vitro reconstructed 3D human skin, keraskin^®^-FT. (**A**) PBS or SP gel (containing FITC-labeled SP) was applied onto the stratum corneum of keraskin^®^-FT. (**B**) Fluorescence images demonstrated the extent of absorption of SP gel. PBS (untreated) or SP gel (containing FITC-labeled SP) was topically applied for indicated duration. Frozen sections of skin tissues were observed under a fluorescence microscope. Scale bar = 1 mm. PBS, phosphate-buffered saline; FITC, fluorescein isothiocyanate; H&E, hematoxylin and eosin.

To evaluate the potential clinical application of SP gel for anti-aging properties, we investigated the ability of SP gel to be absorbed into the stratum corneum (SC), the outermost layer of skin, and enter into the epidermis and dermis. To address this aspect, SP gel containing 5 μg/mL of fluorescein isothiocyanate (FITC)-labeled SP was applied topically to a reconstructed 3D human skin, keraskin^®^-FT (Figure 4A). As shown in Figure 4B, after 1 h of application, the skin sections treated with FITC-labeled SP-containing gel demonstrated a significant green fluorescence throughout the epidermis, along with diffused fluorescence in the dermal tissue, suggesting the ability of SP gel to be absorbed into the SC and become internalized into the epidermis and dermis. After 6 and 24 h, a yet more prominent green fluorescence was observed in the epidermis and dermis of skin sections treated with SP gel. In contrast, no significant fluorescence was observed when only PBS was applied topically.

### 2.5. Melanogenic Effect of SP Gel in a Keratinocyte-Melanocyte Co-Culture System

**Figure 5:**
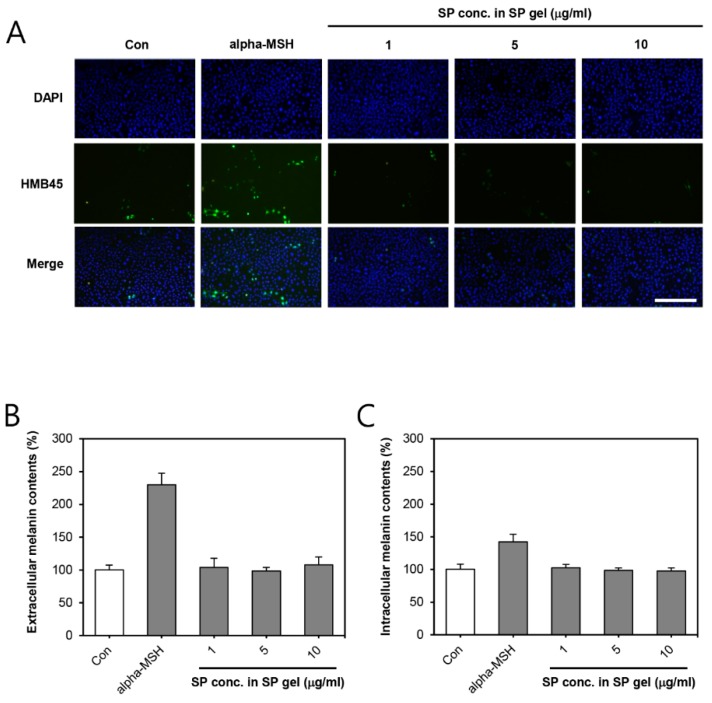
Effect of SP gel on melanogenesis in a keratinocyte-melanocyte co-culture system. (**A**) For immunofluorescence analysis of melanosome, co-cultured cells were treated with an increasing dose of SP gel (1–10 μg/mL) for 72 h, and then fixed and analyzed by immunofluorescence-labeling with a human anti-HMB-45 antibody, finally incubating with fluorescein-conjugated mouse IgG. Nuclei were labeled with DAPI. Scale bar = 500 μm. (**B**,**C**) Quantification of the effect of SP gel on melanin production in the co-culture system. Following incubation with increasing concentrations of SP gel (1–10 μg/mL) for 72 h, the extracellular (B) and intracellular (C) levels of melanin were determined separately by measuring the absorbance at 405 nm. PBS or alpha-MSH was used as a negative or positive control, respectively. All values are represented as mean ± SD from three independent experiments. HMB, human melanoma black; IgG, immunoglobulin G; DAPI, 4′,6-diamidino-2-phenylindole; MSH, melanocyte-stimulating hormone.

After successfully co-culturing melanocytes and keratinocyte (1:5 ratio), the melanogenic effect of SP gel in the co-culture system was investigated. We demonstrated that accumulation of melanosome was not increased by SP gel containing 1–10 μg/mL of SP (Figure 5A), whereas alpha-melanocyte-stimulating hormone (MSH), a positive control, significantly increased melanosome accumulation. To confirm the effect of SP gel on melanin synthesis, the extracellular or intracellular melanin content was quantified in the presence of SP gel in the co-culture system. Figure 5B shows that SP gel containing 1–10 μg/mL of SP did not enhance melanin synthesis relative to PBS-treatment to cells, whereas alpha-MSH strongly increased melanin content in the co-culture system.

## 3. Discussion

SP, an undecapeptide that belongs to the tachykinin family of peptides, has a preferential affinity to the neurokinin-1 receptor (NK-1R) [21]. After binding to NK-1R, SP induces many wound-healing signals, such as cell growth [15,22], collagen synthesis [17,23], and regulation of anti-inflammation [24,25,26]. The wound-healing ability of SP suggests its potential application as an anti-aging material, via collagen production and inflammation inhibition. However, the use of SP has been limited due to its low stability [19,27]. We previously reported a novel formulation of SP, namely SP gel, which elicited stronger wound-healing activities than SP alone, with improved stability [20]. These data together suggest that application of SP gel may indeed enhance the anti-aging effects of SP alone on human skin. However, the anti-aging effects of SP gel have not yet been demonstrated on human skin. Therefore, the present study aimed to determine the anti-aging capability of SP gel for use as a cosmetic ingredient.

In the present study, SP gel significantly increased the expression of type I procollagen, which is the most abundant protein in skin connective tissues [28] (Figure 2A). In general, type I procollagen is mostly degraded by MMPs, which is a family of zinc-requiring endo-proteases, capable of degrading all components of the ECM [29]. In particular, MMP-1 initiates the degradation of type I procollagen [30]. The level of MMP-1 was previously suggested to be directly related to TIMP-1. As a tissue inhibitor of metalloproteinase 1, TIMP-1 indirectly influences ECM-dependent signal transduction in several tissues by inhibiting MMP-1 [31]. In this study, SP gel significantly inhibited the expression of MMP-1 in HDFs (Figure 2B), and mostly enhanced the expression of TIMP-1 (Figure 2C). Collectively, the results suggest the increased level of type I procollagen, caused by SP gel, to possibly be influenced by the inhibition of MMP-1, which in turn, may be correlated with the up-regulation of TIMP-1 in SP gel-treated HDFs. Additionally, the effect of SP gel on collagen synthesis may be explained in terms of the anti-inflammatory effect of SP gel. Inflammation activates the transcription of various factors that degrade metalloproteinases leading to abnormal collagen degradation and non-functional ECM component accumulation [6]. Inflammatory reactions are usually induced by pro-inflammatory cytokines (e.g., IL-1 alpha and IL-6) [32,33], and are known to be inhibited by anti-inflammatory cytokines (e.g., IL-4, IL-10 and TGF-beta 1) [34,35]. Our present results indicate a significant reduction of inflammatory reactions by SP gel in SDS-stimulated HEKs (Figure 3), hence implying that the anti-inflammatory effects of SP gel may have positively influenced collagen synthesis. Therefore, SP gel, as an attractive material with dual function of collagen synthesis and anti-inflammatory effect, has the potential to demonstrate better anti-aging efficacy than conventional agents in clinical practice.

In general, collagen synthesis occurs in HDFs inside the skin [28,36]. Therefore, SP gel should have high skin penetration capacity in order to achieve its anti-aging effects. We, therefore, sought to examine whether SP gel is able to penetrate into a reconstructed 3D human skin model. After 1 h of topical application, SP gel crossed the SC and accumulated in the epidermis. Little accumulation in the dermal tissue was also observed, suggesting that SP gel can be absorbed into the skin (Figure 4B). After 24 h, along with accumulation in the epidermis, strong green fluorescence was observed in the dermis as well, indicating the presence of SP gel there. Otherwise, after 1 h, weak green fluorescence for SP-only treatment was observed in the dermis, but not after 6 h (Figure S2). This means that SP was degraded in the culture conditions of keraskin^®^-FT, suggesting that the degraded SP rapidly penetrated the keraskin^®^-FT and escaped to the medium within 6 h. Although the mechanism by which SP gel gets internalized into the skin is not yet clear, it seems to be related to its characteristic molecular structure. Many amphipathic peptides possess skin absorption abilities [37,38,39], which might mean that the amphipathic characteristics of peptides are the major factors for skin absorption. Indeed, amphipathic peptides have been shown to be taken up by mammalian cells via a non-endocytic mechanism [40,41,42]. The latter, via energy-independent pathways, is initiated by interaction between amphipathic peptides and host membranes, followed by diverse mechanisms that include inverted micelle formation, pore formation, the carpet-like model, and the membrane thinning model [43]. Since SP belongs to a group of small amphipathic peptides, which bind to G-protein coupled receptors [44,45], it can be absorbed into the skin. In addition, a surfactant component, polysorbate 80, present in SP gel, may help to improve its skin absorption. As a known enhancer of skin absorption, the non-ionic surfactant polysorbate 80 contains a long hydrocarbon chain and ethylene oxide, which impart both lipophilic and hydrophilic characteristics to the compound [46]. Therefore, based on the previous findings from several studies, SP gel may be suggested to be absorbed effectively into the skin layer.

In general, components associated with the growth of skin cells are likely to cause skin pigmentation. Several growth factors have the potential to produce skin pigmentation [47,48]. SP gel, which has the effect of increasing the growth of skin cells [20], might as well lead to skin pigmentation, similar to that caused by growth factors. It has been reported that SP increases melanin contents and induces the pigmentation process in human melanocytes [49]. However, Ping et al. [50] showed that SP suppresses melanogenesis in a mouse melanoma cell line, B16-F10 cells, indicating that its potential mechanism may be associated with the inhibition of melanin synthesis pathway, similar to p38 mitogen-activated protein kinase (MAPK) and microphthalmia-associated transcription factor (MITF). These previous reports on pigmentation induced by SP are controversial. The different results can be attributed to the instability of SP. In order to accurately determine whether SP gel induces pigmentation in human skin, we examined its effect on melanogenesis in a keratinocyte-melanocytes co-culture system. In particular, the human keratinocyte-melanocyte co-culture system provides an environment similar to human skin [51], and we applied SP gel to the co-culture system to see its pigmentation effect. In normal co-culture conditions, SP gel was found not to enhance melanosome accumulation (Figure 5A) and melanin synthesis (Figure 5B), thus indicating that SP gel does not induce pigmentation in human skin.

Our results provide evidence in support of the benefits of SP gel as a cosmetic ingredient in anti-aging material. However, additional studies would be required to further clarify the anti-aging effects of SP gel. The present study demonstrates that the anti-aging properties stimulated by SP gel may be directly applied to clinical research. Accordingly, we are now working on confirming the clinical effect of SP gel with respect to anti-aging properties. Additionally, we also plan to investigate the anti-inflammatory effects and clinical toxicity of SP gel in keraskin^®^-FT and human skin, respectively. Based on our present results, SP gel does not seem to cause cell membrane damage and skin irritation in human cells and on reconstructed 3D human skin (Figure 1). Nevertheless, we will perform a primary irritation test, using SP gel on human skin, in the future.

In conclusion, our in-vitro study demonstrated that SP gel treatment could increase type I procollagen levels, correlated with the regulation of MMP-1 and TIMP-1, as well as anti-inflammatory effect. Moreover, we demonstrated the increased skin absorption capability of SP gel, without causing skin pigmentation. Collectively, our findings suggest SP gel to be possibly used as an effective and safe cosmetic ingredient with anti-aging functions.

## 4. Materials and Methods

### 4.1. Materials

All reagents were purchased from Sigma-Aldrich (Louis, MO, USA) and were used as received, unless otherwise indicated. Antibodies against DAPI and melanoma marker (HMB45) were purchased from Vector Laboratories (Burlingame, CA, USA) and DAKO (Carpinteria, CA, USA) respectively. Synthetic SP (RPKPQQFFGLM-NH2) and FITC-labeled SP (FITC-SP) were synthesized and purified to >95% by Anygen (Gwangju, Korea). The enzyme-linked immunosorbent assay (ELISA) kits were purchased from R&D Systems (Minneapolis, MN, USA). Human epidermal keratinocytes (HEKs) and human melanocytes (HMs, SK-MEL-28; ATCC, VA, USA) were cultured in keratinocyte growth medium (KGM; Lonza, Walkersville, MD, USA), and human dermal fibroblasts (HDFs) were cultured in fibroblast growth medium (FGM; Lonza).

### 4.2. SP Gel Treatments

SP gel was produced according to a previous report [52]. It (20 μg/mL of SP in SP gel) was diluted in PBS before being used for treatment of the samples. To determine SP gel-induced toxicity, viability, collagen-increasing effects, and melanogenesis, various concentrations of SP gel (1–10 μg/mL SP in SP gel) were applied to these experiments. In addition, SP gel containing 5 μg/mL of SP or FITC-SP was used for the detection of anti-inflammatory or skin absorption effects of SP gel, respectively.

### 4.3. Isolation and Culture of HEKs and HDFs

We isolated primary HEK and HDF cells from human foreskin biopsies, provided by Chung-Ang University Hospital in Korea [IRB no. C2014234(1431)]. The biopsy samples were washed thrice with PBS solution, and the blood vessels and subcutaneous fatty tissue excised using a sharp blade. The resulting tissue sheets were then cut into small sections and transferred to 0.5% Dispase II solution, followed by incubation at 37 °C for 2 h. The epidermis and dermis were removed using forceps, followed by incubation with 0.05% trypsin diluted in PBS for 30 min. The samples were then transferred to 0.2% collagenase solution and incubated at 37 °C for 2 h. Trypsin and collagenase activity was then stopped by the addition of 10% fetal bovine serum, and the tissue fragments were filtered through a cell strainer and washed in PBS. The resulting HEK and HDF cells were seeded onto plastic dishes and cultured in KGM and FGM, respectively.

### 4.4. Cell Membrane Damage Assay

To analyze the damaging effects of SP gel on the cell membrane, HDFs were cultured in 96-well plates (3 × 10^3^ cells/well) in FGM. After 24-h incubation, cells were treated with SP gel (1–10 μg/mL of SP in SP gel) and incubated for another 24 h. LDH-based assays (Roche Applied Science, Mannheim, Germany) were performed, according to the manufacturer’s instructions, to further test the effects of SP on cell membrane damage. We treated positive controls with 1 % Triton X-100 and set LDH release to 100%. Relative LDH release was calculated, using intact cells, from the ratio of released LDH over total LDH. LDH release <10% was considered to be non-toxic. Three independent experiments were performed to confirm our findings.

### 4.5. In-Vitro Skin Irritation Test

Reconstructed human epidermal tissues (Keraskin^®^-FT) were purchased from Biosolution Co., Ltd. (Seoul, Korea). In accordance with previous studies [53], the tissues were conditioned by overnight incubation on the day of receipt. After pre-incubation for 24 h, they were transferred to a six-well plate supplemented with fresh medium, and topically treated with 100 μL of negative control (PBS), positive control (5% SDS), and SP gel (1–10 μg/mL of SP in SP gel) for 24 h at 37 °C. The tissues were thoroughly washed with PBS thereafter, and transferred to fresh medium. After culturing for 48 h, cell viability assay was conducted by transferring the tissues to a 24-well plate containing MTT medium (0.3 mg/mL), followed by 3-h incubation. Blue formazan was extracted with isopropanol, and its optimal density was confirmed at 570 nm using a VersaMax tunable microplate reader (Molecular Devices, Sunnyvale, CA, USA). Relative cell viability was calculated for each tissue, as a percentage of the mean of negative control tissue. For histological evaluation, tissues were fixed in 4% formaldehyde, and processed for embedding in paraffin. Subsequently, 5-μm vertical sections were cut and stained with hematoxylin and eosin (H&E) for examination under a BX41 light microscope (Olympus, Tokyo, Japan). At least three independent experiments were performed.

### 4.6. Measurement of Type 1 Procollagen, MMP-1, and TIMP-1

Levels of type I procollagen, MMP-1, and TIMP-1in HDFs were quantified using the procollagen Type I C-peptide (P1P) EIA kit (Takara Bio, Inc., Otsu, Japan), and human MMP-1 and TIMP-1 ELISA kits (R&D systems), respectively. The HDFs were seeded at a density of 3 × 10^3^ cells/well. After 48 h, the cells were treated with PBS (negative control) or various concentrations of SP gel (1–10 μg/mL) for 24 h. The levels of type I procollagen, MMP-1, and TIMP-1 in the culture media were measured according to the manufacturer’s instructions. Results for each group were expressed as the percentage of the mean of the negative control group.

### 4.7. Measurement of IL-1 Alpha, -6, -10, and TGF-Beta 1

HEKs were seeded at a density of 3 × 10^4^ cells/well. After 48 h, the cells were treated with PBS (negative control), 15 ng/mL of SDS, or SP gel (5 μg/mL SP in SP gel) for 24 h. The cell supernatants were collected for the detection of inflammatory cytokines such as IL-1 alpha, -6, -10, and TGF-beta 1. The concentration of cytokines in the supernatants was determined by ELISA kits, according to the manufacturer’s instructions. Concentrations in the samples were calculated with reference to the corresponding standard curves and were expressed for each group as a percentage of the mean of the negative control group. At least three independent experiments were performed.

### 4.8. Skin Absorption of SP Gel in the Reconstructed Human Epidermal Tissues

Topical skin absorption of SP gel (5 μg/mL of SP in SP gel) was evaluated using the reconstructed human epidermis, keraskin^®^-FT. The SP gel treatment scheme is shown in Figure 4A. Briefly, 100 μL of SP gel, containing FITC-SP, was topically applied on the keraskin^®^-FT, and tissue sections were fixed overnight in ice-cold 4% formaldehyde. Subsequently, tissue samples were washed in PBS and then immersed in a 4.5% sucrose solution for 24 h. This was followed by dehydration of the samples in 30% sucrose until deposition occurred. We used a freezing microtome (Leica, Wetzlar, Germany) to produce five-micron-thick cryo-sections, which were then imaged using a fluorescence microscope (Nikon, Tokyo, Japan).

### 4.9. Co-Culture and Immunofluorescence

HEKs or HMs were cultured in KGM. They were harvested by treatment with trypsin/EDTA, and were re-suspended in KGM. Viable cells were seeded at 2.5 × 10^4^ cells/well (for HEKs) and 5 × 10^3^ cells/well (for HMs), simultaneously in 12-well plates. Co-cultures of HEKs and HMs (HEKs-HMs) were maintained in KGM, the initial seeding ratio of HEKs to HMs being 5:1. After three days, fresh medium containing PBS (negative control), alpha-MSH (150 nM), or SP gel (1–10 μg/mL of SP in SP gel) was added when HEKs-HMs were about 60% confluent. Two days later, activation of HMs was detected by indirect immunofluorescence images. DAPI (100:1 dilution), specific for cell nuclei, and HMB 45 (1:100 dilution), specific for gp100 (to detect stage II-IV melanosomes), were applied to HEKs-HMs at 4 °C for 18 h. HMB 45 was observed using an appropriate secondary antibody: Fluorescein mouse IgG (H + L) at 1:100 dilution with 5% goat serum at 25 ± 2 ℃ for 2 h. Fluorescence was observed, and data analyzed using a fluorescence microscope (Leica).

### 4.10. Extracellular and Intracellular Melanin Content

Melanin content was determined and quantified using a previously described method, with slight modifications [54]. HEKs-HMs were co-cultured in KGM for three days and treated thereafter with PBS (negative control), alpha-MSH (150 nM), or SP gel (1–10 μg/mL of SP in SP gel) for 48 h. The cultured cells or media were harvested, and pellets were dissolved in 1N NaOH containing 10% dimethyl sulfoxide (DMSO) at 80 °C for 1 h. Melanin content was measured using an absorbance reader at 475 nm, and it was eventually normalized to the cellular protein concentration. At least three independent experiments were performed.

### 4.11. Statistical Analysis

Data are reported as mean ± SD and analyzed with the Student’s *t*-test. *p* < 0.05 was considered statistically significant.

## Supplementary Materials

The following are available online at https://www.mdpi.com/1422-0067/20/18/4453/s1.

## Abbreviations

LDH	Lactate dehydrogenase
MTT	3-(4,5-dimethylthiazol-2-yl)-2,5-diphenyl tetrazolium bromide
SDS	Sodium dodecyl sulfate
MMP-1	Matrix metalloproteinase-1
TIMP-1	Tissue inhibitor of metalloproteinase-1
alpha-MSH	Alpha-melanocyte stimulating hormone
DAPI	4′,6-diamidino-2-phenylindole
HMB 45	Human melanoma black
NK-1R	Neurokinin-1 receptor
