# Supplementary material for: Anti-Aging Potential of Substance P-Based Hydrogel for Human Skin Longevity
Source: Int J Mol Sci. 2019 Sep 10;20(18):4453. doi: 10.3390/ijms20184453 (PMC6770478; doi:10.3390/ijms20184453)
Supplement: Supplementary file 1 [file ijms-20-04453-s001.pdf]

Supplemental information

Supplementary materials and methods

In vitro skin irritation test of SP alone

Keraskin®-FT was prepared using the same methods as in the original paper, before treating topically with 100 μl of SP (1-10 μg/ml SP in PBS) for 24 h at 37 ℃. After washing with PBS and culturing for 48 h, the MTT assay was performed. For histological evaluation, tissue sections were stained with H&E. At least two independent experiments were performed.

Skin absorption of SP alone in the reconstructed human epidermis tissues

Topical skin absorption of SP alone (5 μg/ml SP in PBS) was evaluated on the reconstructed human epidermis tissue, keraskin®-FT. SP alone (100 μl) containing FITC-SP in PBS was added topically to the keraskin®-FT, and tissue sections were fixed overnight in ice cold 4% formaldehyde after the indicated time. Thereafter, these tissue samples were washed and immersed in 4.5% sucrose solution for 24 h followed by dehydration in 30% sucrose until deposition occurred. Cryosections (5 μm thickness) were obtained using a freezing microtome (Leica, Wetzlar, Germany). The skin sections were imaged under a fluorescence microscope (Nikon, Tokyo, Japan).

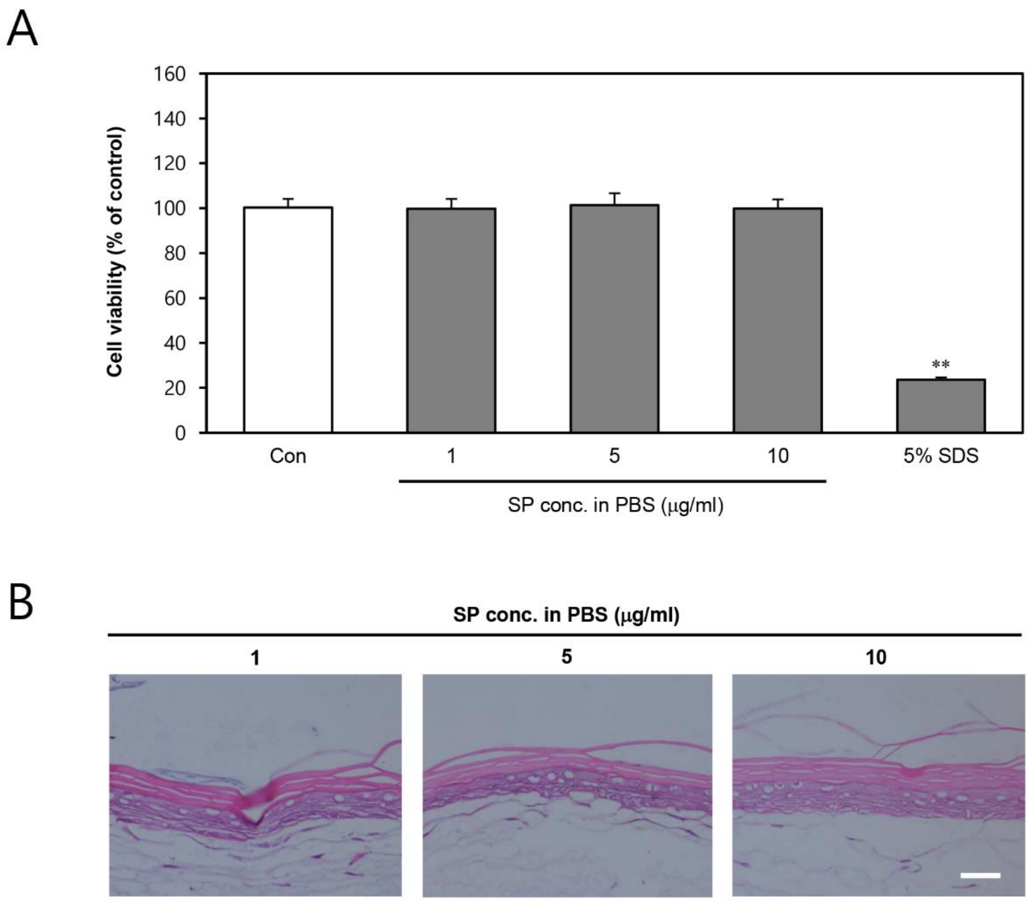

Figure S1. Effect of SP alone on the viability of in-vitro reconstructed 3D human skin (**A**) For tissue viability with SP alone, keraskin®-FT was treated with PBS (Con; control), SP alone (1-10 mg/ml), or 5% SDS for 24 h, and tissue viability was determined by MTT assay. (**B**) Histological examination was analyzed by evaluation of H&E staining. Values represent mean ± SD of two independent experiments. Each value was compared with that of the control using student's *t*-test (**P < 0.01). Scale bar = 500 μm. MTT, 3-(4,5-dimethylthiazol-2-yl)-2,5-diphenyl tetrazolium bromide; PBS, phosphate buffered saline; SDS, sodium dodecyl sulfate.

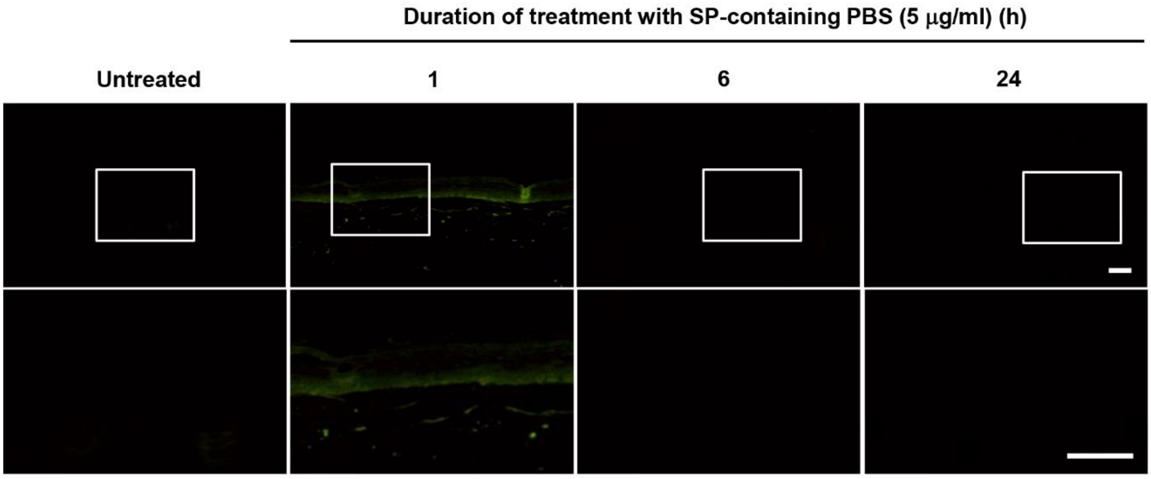

Figure S2. Skin absorption of SP alone into in-vitro reconstructed 3D human skin, keraskin®-FT. Fluorescence images demonstrate the skin absorption of SP alone. PBS (untreated) or SP alone (containing FITC-labeled SP in PBS) was topically applied for the indicated times. Frozen sections of skin tissues were obtained and observed by fluorescence microscopy. Scale bar = 1 mm. PBS, phosphate-buffered saline; FITC, fluorescein isothiocyanate.
